# Primary Middle Meningeal Artery Embolization for a Chronic Subdural Hematoma After Non-Accidental Trauma in a Child: A Case Report

**DOI:** 10.7759/cureus.26399

**Published:** 2022-06-28

**Authors:** Saisree Ravi, Sohum Desai, Ameer E Hassan, Wondwossen G Tekle

**Affiliations:** 1 Department of Neurology, University of Texas Rio Grande Valley School of Medicine, Harlingen, USA; 2 Department of Neurosurgery, University of Texas Rio Grande Valley School of Medicine, Harlingen, USA

**Keywords:** endovascular surgical repair, chronic subdural hematoma (csdh), non-accidental trauma, pediatric head trauma, mma embolization

## Abstract

Chronic subdural hematoma in children can be pathognomonic of abusive head trauma. Treatment options for these range from observation to surgical evacuation depending on clinical circumstance and presenting features, which can include mental status changes, headaches, focal neurologic deficits, or asymptomatic presentation. Standalone endovascular treatments represent an area of growing interest in the adult population as an effective treatment modality. However, embolization as a singular treatment approach has not been reported in the pediatric population. We report the first case of stand-alone middle meningeal artery (MMA) embolization of a chronic subdural hematoma as a sequela of abusive head trauma in a two-year-old child, resulting in complete resolution on non-contrast CT head at six months post embolization.

## Introduction

Chronic subdural hematomas are known sequelae of non-accidental trauma in children. Management options for them include observation with serial imaging in asymptomatic patients, subdural punctures in symptomatic children with open fontanelles, burr hole drainage, craniotomy, and even subdural-peritoneal shunt in some recurrent cases [1]. Middle meningeal artery (MMA) embolization is a developing therapy that has been shown to aid the resorption of chronic subdural hematomas in the adult population and aid in preventing recurrence [2]. Its use in children has been reported only once in the literature, primarily in an adjunctive fashion to surgical therapy [3]. We review the literature and report the first case of utilizing MMA embolization as a standalone treatment to address chronic subdural hematoma after non-accidental trauma.

## Case presentation

**Figure 1:**
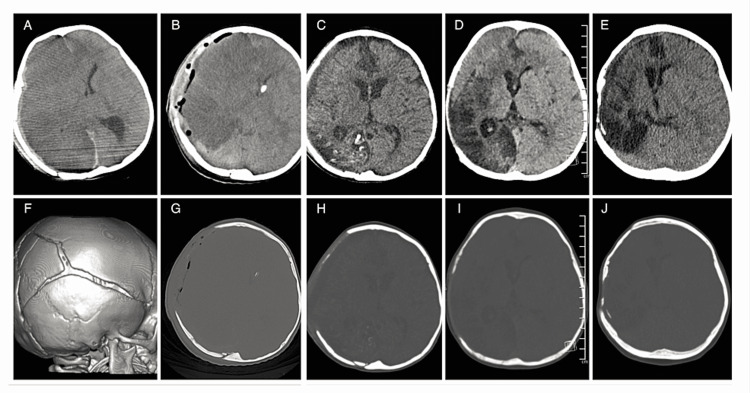
Non-contrast head CTs at successive time intervals pre and post middle meningeal artery embolization (A) Preoperative axial non-contrast CT head demonstrates an acute right-sided subdural hematoma, large right-sided hemispheric infarction, and significant midline shift; (F) 3D reconstruction of the preoperative scan demonstrates multiple skull fractures crossing sutures. (B) Postoperative CT demonstrating craniectomy defect with left-sided ventricular drain placement. (C) CT obtained prior to discharge to inpatient rehabilitation facility. (D) CT obtained at three month outpatient follow-up demonstrating formation of the right frontal chronic subdural hematoma deforming the frontal lobe. There is extensive cortical and subcortical encephalomalacia of the right hemisphere. (E) Follow up CT obtained six months after MMA embolization demonstrating complete resolution of the right frontal chronic subdural hematoma. (G-J) Axial non-contrast CT head corresponding to same slices seen on (B-E) but with windowing optimized for bone to demonstrate progressive ossification over the craniectomy defect between each imaging interval.

A two-year-old female who was the apparent victim of non-accidental trauma presented to our institution comatose with a dilated non-reactive right pupil. A CT was obtained which demonstrated an acute right-sided subdural measuring 5 mm in thickness associated with a 10 mm midline shift in addition to multiple skull fractures (Figure 1A, 1F). She was taken to the operating room where she underwent a right-sided decompressive craniectomy with the evacuation of the acute subdural hematoma and placement of a left-sided ventriculostomy drain (Figure 1B). Her bone flap was swabbed and stored in a nitrogen freezer per institutional protocol. Postoperatively, she was transferred to the pediatric intensive care unit (PICU) and treated for cerebral edema with sedation, cerebrospinal fluid (CSF) drainage, and hyperosmolar therapy. Intracranial pressure began to normalize and these interventions were gradually de-escalated. On postoperative day 5, she began to open her eyes and localize with her right upper extremity and was subsequently extubated on postoperative day 8. She was eventually discharged to inpatient rehabilitation approximately 1 month later (Figure 1C).

**Figure 2:**
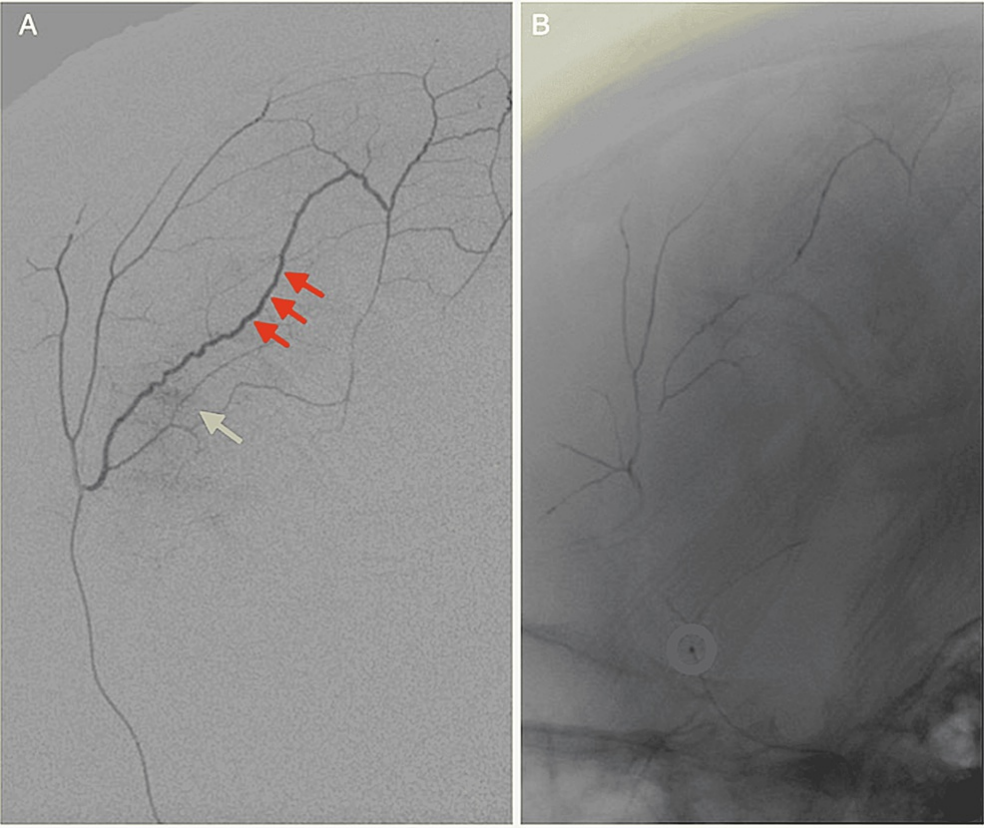
Microcatheter positioning during middle meningeal artery embolization (A) Lateral projection demonstrates selective microcatheter run demonstrates a dilated anterior division of the right middle meningeal artery seen with a red arrow and associated contrast blush seen in yellow arrows of the subdural membrane. (B) Final position of the microcatheter prior to completion of the embolization.

Three months after her index surgery, she returned to the clinic for evaluation of cranioplasty. On exam, she continued to have third nerve palsy but was able to crawl independently and feed herself. Her previously-stored bone flap had been discarded due to positive culture results for methicillin-sensitive Staphylococcus aureus. A CT head without contrast was then obtained for the purposes of planning a custom cranioplasty implant. This demonstrated interval development of right frontal chronic subdural hematoma (Figure 1D) as well as significant heterotopic ossification over the prior craniectomy site (Figure 1I). Given the development of a chronic subdural hematoma and the desire to avoid disturbing the ongoing bony remodeling (Figure 1G-1I), she was referred to the neuro-endovascular service for middle meningeal artery embolization under general anesthesia. Under fluoroscopic guidance, the 5-French multi-purpose drainage (MPD) catheter was advanced into the right external carotid artery, then a Duo 156 microcatheter was advanced over Synchro-2 microwire and selectively catheterized to the middle meningeal artery (Figure 2A); 0.35 mL of dimethyl sulfoxide (DMSO) and the same amount of Onyx 18 were infused to fill the dead space. Then, a total of 0.2 mL of Onyx 18 was infused until there was significant reflux with multiple breaks with no further antegrade flow, and the procedure was aborted with no residual MMA appreciated (Figure 2B). The patient then underwent follow-up at six months postembolization with a non-contrast CT head which demonstrated near resolution of the chronic subdural hematoma (Figure 1E). Neurologically, at the last follow-up, she continues to have a third nerve palsy on the right but is now able to walk short distances with a circumducting gait unassisted and communicates through simple sign language.

## Discussion

The optimal management strategy for chronic subdural hematoma in children is not well defined. When operative intervention is indicated, options such as transfontanelle needle aspiration have up to a 74% recurrence rate [1]. While other procedures such as burr hole drainage and craniotomy are more definitive, many patients still develop recurrence and some even require subdural-peritoneal shunts, requiring long-term follow-up for a permanent implant. MMA embolization is an evolving treatment option for chronic subdural hematoma. As of the writing of this paper, there are 15 clinical trials actively recruiting patients [3-5]. Embolization is theorized to stop a self-propagating cycle beginning with inflammation followed by angiogenesis of leaky capillaries, resulting in exudate formation and ultimately hematoma expansion [2]. It has not been well studied in the pediatric population, with only two prior case reports in the literature to the best of our knowledge, as further described in Table 1 [3,4]. Our case bears a resemblance to the study by Farber et al. on a child who developed a chronic subdural from non-accidental trauma. However, unlike in their case, our patient’s chronic subdural was treated entirely with embolization without concurrent hematoma evacuation [3]. Shigematsu et al. report a single case of standalone MMA embolization for chronic subdural hematoma occurring secondary to antiplatelet and anticoagulant medication use while she was undergoing preparation for an eventual heart transplant [4]. This case, like ours, does demonstrate the feasibility of achieving complete resorption of chronic subdural, albeit for a completely different diagnosis. From a technical standpoint, our preference is a transfemoral bi-axial set up with a short guide catheter and microcatheter. Unlike in adult patients, we feel distal access catheters (DAC) are redundant given the lack of vessel tortuosity. Furthermore, the small-caliber vessels in children that are prone to spasms would make the addition of a DAC problematic. Another unique aspect of this case was that continued re-ossification within the craniectomy site (Figure 1H-1J) was not interrupted by MMA embolization. Immature dura contains cells that aid in calvarial reossification that are not present in adults [6]. This, fortunately, allowed us to continue managing the patient’s skull defect as ossification continues. However, there are certain shortcomings to this embolization technique for the pediatric population. The guide MPD catheter used in this case had a length of 90 cm and the Duo microcatheter had a length of 156 cm, which were the shortest available options. Yet, these were still quite long and not completely apt for the height of the patient, presenting a drawback in the mechanism of this technique when applied to children. Thus, further investigation is required to explore options that optimize catheter length and size according to the age and height of pediatric patients.

**Table 1:** Characterization of literature reporting middle meningeal artery embolization in pediatric chronic subdural hematomas A literature search reveals two prior case reports with MMA embolization in pediatric patients, with one report of stand alone MMA embolization secondary to cardiac diagnosis [4] and one report of adjunct embolization following subdural evacuation [3].

Authors	Age of patient	Intervention approach	Outcome
Farber et al. [3]	18 months male	Craniotomy for subdural evacuation followed by MMA embolization 6 weeks later	Size and density of subdural hematoma decreased on head CT at 2 weeks, 3 months, and 6 months post embolization with subsequent normal development
Shigematsu et al. [4]	5 months female	MMA embolization for chronic subdural hematoma secondary to dual antiplatelet and antithrombotic therapy, with heart transplant due to dilated cardiomyopathy following embolization	Successful post embolization heart transplantation with significant improvement of chronic subdural hematoma and neurologically asymptomatic with subsequent normal development

## Conclusions

Middle meningeal artery embolization can be used as a standalone treatment for subdural hematomas that occur after non-accidental trauma in children. This case demonstrates that embolization holds significant potential as an effective modality for complete resolution of subdural hematomas in children, as evidenced by imaging on follow-up. However, further studies are needed to elucidate other aspects in terms of tailoring the treatment approach and tools according to age and height in the pediatric population.
